# Attainment of polarity promotes growth factor secretion by retinal pigment epithelial cells: Relevance to age-related macular degeneration

**DOI:** 10.18632/aging.100111

**Published:** 2009-12-27

**Authors:** Shozo Sonoda, Parameswaran G. Sreekumar, Satoru Kase, Christine Spee, Stephen J Ryan, Ram Kannan, David R Hinton

**Affiliations:** ^1^ Department of Ophthalmology, Keck School of Medicine of the University of Southern California, Los Angeles, CA 90089, USA; ^2^ Department of Pathology, Keck School of Medicine of the University of Southern California, Los Angeles, CA 90089, USA; ^3^ Arnold and Mabel Beckman Macular Research Center at the Doheny Eye Institute, Los Angeles, CA 90033, USA; ^4^ Department of Ophthalmology, Kagoshima University, Graduate School of Medical and Dental Sciences, Kagoshima, Japan; ^5^These two authors contributed equally to this work

**Keywords:** retinal pigment epithelial cell, cell polarity, VEGF-A, PEDF, BMP-4, age-related macular degeneration

## Abstract

The antiangiogenic and neurotrophic growth factor, pigment epithelial derived factor (PEDF), and the proangiogenic growth factor, vascular endothelial growth factor-A (VEGF), are released from retinal pigment epithelial (RPE) cells where they play a critical role in the pathogenesis of age-related macular degeneration (AMD). Since RPE polarity may be altered in advanced AMD,    we studied the effect of polarization of differentiated, human RPE monolayer cultures on expression and secretion of PEDF and VEGF. Polarized RPE demonstrated apical microvilli, expression of tight junction proteins, apical localization of Na/K- ATPase, and high transepithelial resistance (490 ± 17 Ω•cm^2^).  PEDF secretion was about 1000 fold greater than that for VEGF in both polarized and non-polarized cultures. Polarization of the RPE monolayer increased PEDF secretion, which was predominantly apical, by 34 fold (p<0.02) and VEGF secretion, which was predominantly basolateral, by 5.7 fold (p<0.02). Treatment of non-polarized RPE cultures with bone morphogenetic protein-4 (BMP-4) had no effect on PEDF or VEGF secretion, but resulted in a dose-dependent >2-fold increase in basolateral VEGF secretion (p<0.05) in polarized cultures.  Our data show that polarity is an important determinant of the level of PEDF and VEGF secretion in RPE and support the contention that loss of polarity of RPE in AMD results in marked loss of neurotrophic and vascular support for the retina potentially leading to photoreceptor loss and blindness.

## Introduction

The retinal pigment epithelium (RPE), strategically located between the light sensitive photoreceptors and the choroid, is a monolayer of highly specialized cells that serves as the outer blood-retinal barrier, selectively transporting biomolecules between the neural retina and choriocapillaris, and secreting factors that protect their health and integrity [1,2]. In the last decade, a number of reports on the utility of *in vitro* cell culture systems for studyingpathophysiology of RPE have appeared (reviewed in [3]). Cell culture models can play an important role in gaining knowledge about native tissue since appropriate RPE function relies on the maintenance of its polarity [3].

Several laboratories have attempted to establish polarized RPE monolayer cultures using Transwell membrane filters in order to mimic the native RPE monolayer [3-7]. Most studies have been performed with a human RPE cell line, ARPE-19, spontaneously transformed using multiple trypsinizations [8-10]. However, it is common for differentiated cells to lose their specialized properties after multiple passages; ARPE-19 cells showed relatively low transepithelial resistance (TER) and depend on highly specific culture conditions for the development of functional tight junctions [11-13]. In a report comparing the barrier properties of ARPE-19, D-407, primary RPE cells from C57Bl/6 mouse, and primary human fetal RPE, only those culture systems with well differentiated monolayers showing high TER (>500 Ω·cm^2^) were found to be suitable for studying growth factor regulation [14]. Among the methods for polarization of human fetal RPE, the method of Hu and Bok [4] has been widely recognized for its differentiated phenotype, and high TER; however, their method requires use of a complex medium including uncharacterized brain extracts.  Recently, a simplified cell culture procedure was developed using human fetal RPE to produce highly differentiated, polarized monolayers that were used to demonstrate asymmetrical polarized secretion of several cytokines [15,16]. Yet, there has been relatively little specific focus on differences between non-polarized and highly polarized human RPE cells from individual donors with respect to the level of growth factor expression and secretion.

Disruption of the equilibrium of secretion from apical and basolateral surfaces of the RPE monolayer is believed to promote a pathological microenvironment, thus contributing to various retinal diseases [5,6,17].  For example, in choroidal neovascularization (CNV), which occurs late during the course of age-related macular degeneration (AMD) [18,19], dysregulated expression of the proangiogenic growth factor, vascular endothelial growth factor-A (VEGF) [20,21], and the neutrotrophic and antiangiogenic growth factor, pigment epithelium derived growth factor (PEDF) [22], is thought to play an important role in the pathogenesis of the disease. The primary insult in the late form of dry AMD (geographic atrophy;GA) appears at the level of RPE and a close relationship between RPE atrophy and secondary choriocapillaris degeneration was reported [23].  Further in GA, it was recently shown that progressive RPE alterations occur in the expression of basolaterally located proteins such as CD63 and MCT3 [24]. Thus, in both late forms of AMD (CNV and GA) there are alterations in RPE polarity that might contribute to an altered growth factor microenvironment.

Several cytokines are known to affect the secretion of VEGF and PEDF [25,26]. In a recent study, it was reported that treatment of non-polarized ARPE-19 cells with Bone morphogenetic protein-4 (BMP-4) increased VEGF synthesis and secretion [27]. BMP-4 plays an important role in RPE development and specification [28,29], is preferentially expressed in RPE in the adult retina [30,31], and is over-expressed in RPE in dry AMD where it may play a role in AMD pathogenesis by induction of RPE senescence [32].  The primary aim of this study was to determine the effect of polarization of RPE on expression and secretion of PEDF and VEGF in the unstimulated state, and after stimulation with BMP-4.

## Results

### Functional and morphological characterization of human polarized RPE cells

**Figure 1.**
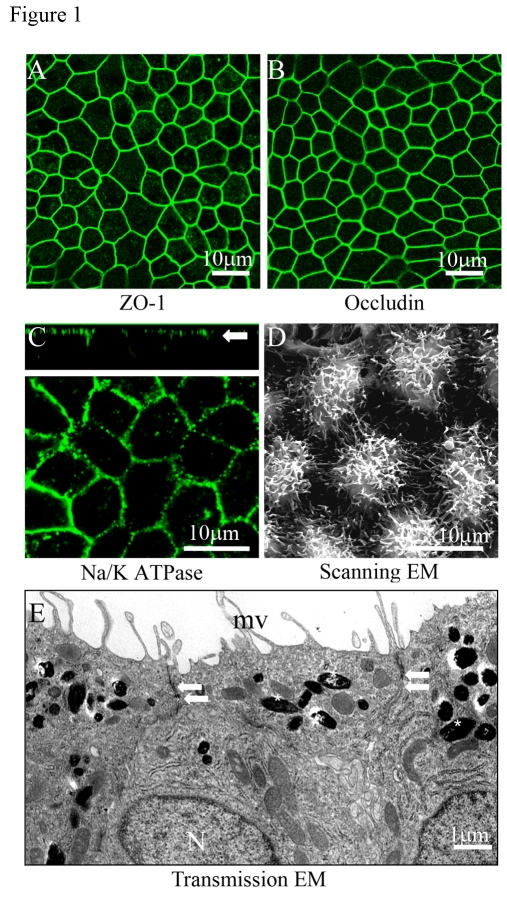
Confocal and electron microscopic characterization of polarized RPE cells. Evidence for tight junction proteins and polarity in fetal RPE cells cultured on Transwell filters for 6 weeks.  (**A, B**) Immunofluorescence staining of tight junction proteins ZO-1 and occludin. (**C**) Localization of Na/K- ATPase to the apical plasma membrane as shown in the confocal vertical (X-Z) section (white arrow). (**D**) Well differentiated apical microvilli observed by scanning electron microscopy (SEM). (**E**) Well developed microvilli (mv), localization of pigment on the apical side (asterisks), nuclei on basal side (N), and presence of tight-junctional complexes (arrows) by transmission electron microscopy (TEM).

As in native tissue, human RPE cells on Transwell filters formed a monolayer, were well pigmented, and were arranged in a regular hexagonal array. Confocal immunofluorescent studies of cultures grown in 1% FBS for one month showed that the intercellular assemblage outlining each cell was positively stained for tight junction protein is ZO-1 and occludin (Figure 1A, B). To establish that the cultured RPE cells exhibit polarity, we stained for the apical marker enzyme Na/K- ATPase. As expected, Na/K- ATPase was localized to the apical plasma membrane of the RPE cells as shown in the confocal vertical (X-Z) section (Figure 1C, arrow). Figure1D shows a scanning electron micrograph of the apical surface of the RPE monolayer with well-developed apicalmicrovilli. Furthermore, transmission electron micrographs show that RPE have basally located nuclei, contain melanin pigment granules that congregate on the apical side of the cytoplasm, and exhibit well-developed tight-junctional complexes and apical microvilli (Figure 1E).

Weekly measurements of TER were made in RPE monolayers maintained in 1% FBS for up to one and a half months. The resistance showed a gradual increase with time and began to plateau at one month. The TER values in polarized RPE cells at one month averaged 490±17 Ω·cm^2^ (mean ± SEM, n=48).

### Significant difference in PEDF and VEGF secretion between nonpolarized and polarized RPE

**Figure 2.**
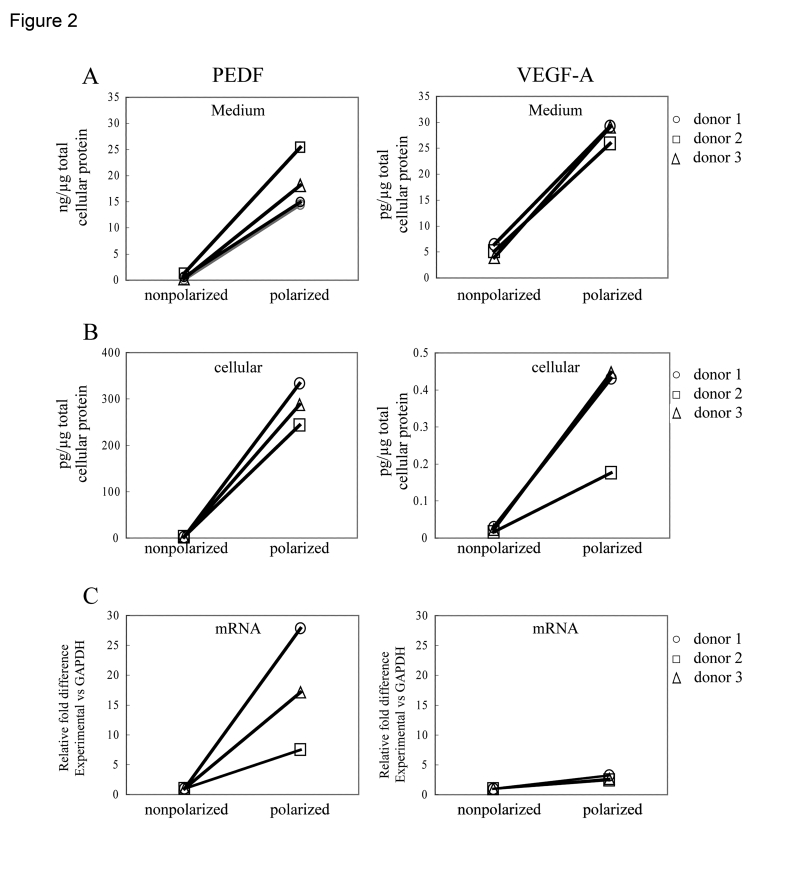
Differences in PEDF and VEGF secretion between nonpolarized and polarized RPE from various donors after 24h. Secretion from the polarized RPE cells represent the sum of experimentally determined apical and basolateral secretion values, normalized for total cellular protein. The total secretion increased 34 fold for PEDF and 5.7 fold for VEGF-A (**A**). Analysis of cellular protein (**B**) and mRNA (**C**) showed that expression in polarized human RPE was higher compared to nonpolarized RPE cells.

Experiments were performed using confluent non-polarized, and confluent polarized RPE cells from the same human donors to determine the influence of polarity on PEDF and VEGF secretion (Figure 2A).  PEDF and VEGF secretion was measured in the supernatants from both nonpolarized, and polarized RPE cells. The secretion from the non-polarized cells represents the total growth factor content in the medium of a 6-well plate, while for polarized cells, the secretion represents the sum of growth factor content in the apical and basolateral medium; in all cases, data have been normalized for total cellular protein. The concentration of PEDF was approximately 1000X greater than that of VEGF-A for both non-polarized and polarized RPE cultures (Figure 2A). For each donor, the amount of secretion of PEDF and VEGF in highly polarized RPE cells was significantly higher (p<0.02) than for confluent, nonpolarized RPE. The amount of VEGF secretion increased 5.7 fold, while that of PEDF was 33.6 times higher for polarized cells than non-polarized cells. Similarly, the PEDF and VEGF cellular content, normalized for total cellular protein, also increased in polarized cells over non-polarized cells by >100-fold for PEDF (p<0.01) and 15-fold (p<0.06) for VEGF (Figure 2B). Cellular mRNA expression of both PEDF and VEGF was also elevated in polarized cells; PEDF mRNA expression was 18 fold higher in polarized vs non-polarized RPE, while VEGF mRNA expression was 2.8-fold higher in the polarized cells (Figure 2C). These data demonstrate that induction of polarity in RPE is associated with increased mRNA expression, increased cellular protein expression, and increased secretion of PEDF and VEGF.

### Polarized secretion of PEDF and VEGF from well differentiated RPE cells

**Figure 3.**
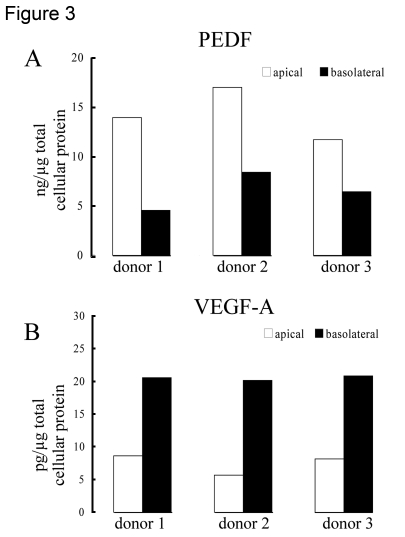
Polarized secretion of PEDF and VEGF in differentiated human RPE cells. Human polarized RPE cells on transwells isolated from 3 different donors preferentially secreted PEDF (**A**) to the apical side of the tissue and VEGF-A (**B**) to the basolateral side. The bars represent average of 2 determinations for each donor with variation in each sample <5%.

The extracellular incubation medium from 3 donors was used to quantify the amount of PEDF and VEGF secreted into the apical *vs* basolateral sides. Human polarized RPE cell grown on Transwell culture membranes secreted PEDF preferentially to the apical side of the tissue (p<0.03) and VEGF to the basolateral side (p<0.01). The mean (± SEM) concentration of PEDF in the apical and basolateral supernatants was 14.2 ± 1.5 ng/μg total cellular protein and 6.5 ± 1.1 ng/μg total cellular protein, respectively (Figure 3A). In contrast, VEGF concentration was 7.5 ± 0.9 pg/μg total cellular protein (mean ± SEM, apical) and 20.6 ± 0.2 pg/μg total cellular protein (mean ± SEM, basolateral), in apical and basolateral supernatants respectively. (Figure 3B). The amount of PEDF secreted into the apical and basolateral supernatants was >1800 times and >300 times higher than that of VEGF-A respectively.

### Cellular distribution of PEDF by confocal immunofluorescence staining

**Figure 4.**
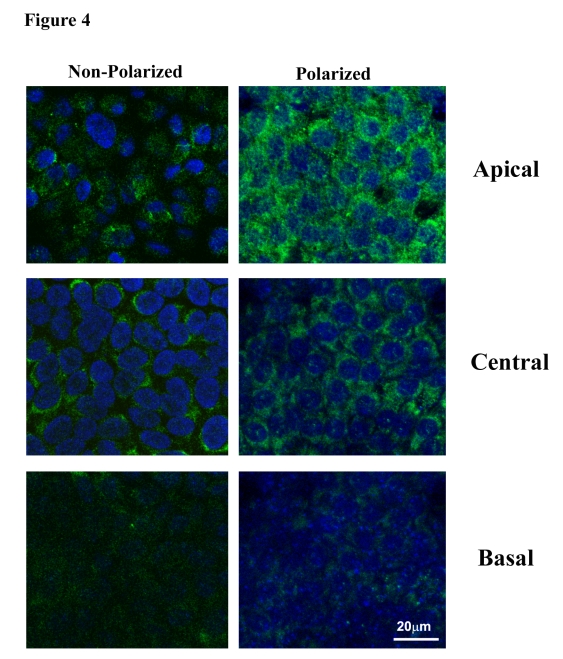
Distribution of PEDF in apical, central and basal regions in nonpolarized and polarized RPE cells by confocal microscopy. Staining for PEDF is more intense in polarized RPE as compared to nonpolarized RPE.  The apical region shows much higher PEDF expression in polarized cells.

Figure 4 shows the confocal immunofluorescent staining for PEDF in nonpolarized and polarized RPE cells. The intensity of PEDF staining was found to be much higher for polarized RPE as compared to nonpolarized RPE.  Further, examination of subcellular distribution in the polarized RPE revealed a progressive increase in  PEDF expression from basal to central to apical regions, with maximal expression seen in the apical region.  This pre-dominant staining in the apical region is consistent with a significantly higher apical secretion shown in Figure 3.

### Cell cycle analysis of polarized and nonpolarized RPE cells

**Figure 5.**
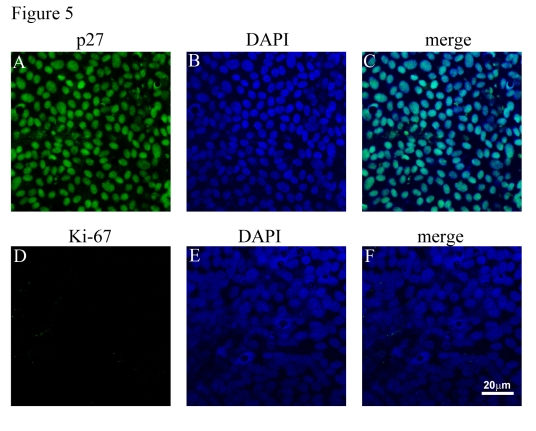
Cell cycle analysis of polarized RPE monolayers. (**A, B, C**) Expression of p27 (green) and its localization to nuclei (blue). (**D, E, F**). Polarized RPE cultures show lack of expression of Ki-67 (green) in the nuclei. Nuclei counterstained blue with DAPI.

It has been reported previously that cellular proliferation/cell cycle can influence the amount of PEDF secretion by human fibroblast-like cells [33,34]. We determined whether cells were in cycle vs cellular quiescence by evaluating the nuclear expression of Ki-67 (cells in cycle) and p27 (cellular quiescence) under three conditions, viz. confluent RPE (condition 1), confluent-quiescent non-polarized RPE (condition 2), and confluent polarized RPE (condition 3). Polarized RPE monolayers showed almost 100% positivity for p27 and barely any cells (<0.1%) positive for Ki-67 indicating that these cells were in a quiescent stage (Figure 5; Table 1). On the other hand, the just confluent non-polarized RPE cells showed an opposite staining pattern with almost 90% of cells positive for Ki-67 and <1% of cells positive for p27 indicating that these cells were in cell cycle (Table 1). To determine whether the differences in growth factor secretion between polarized and non-polarized confluent cells were due to differences in cell cycle, we also evaluated confluent-quiescent cultures (condition 2; cells cultured an additional 7 days  in 1% FBS) for their expression of Ki-67 and p27, and their levels of growth factor secretion. Confluent-quiescent, non-polarized cultures were predominantly quiescent with <5% Ki-67 positivity and almost 60% p27 positivity (Table 1); a pattern that was close to that of polarized RPE monolayers (Table 1). While confluent polarized RPE showed 33.6 fold  increased PEDF secretion compared to confluent non-polarized RPE, the confluent-quiescent RPE showed only a two fold  increase (2.20 ± 0.21, mean ± SEM) compared to confluent non-polarized RPE cells. These results provide strong support for the contention that polarization, rather than quiescence, largely contributes to increased PEDF secretion found in confluent polarized monolayers.

**Table 1.** Relative proportion of Ki-67 and p27 positive cells in human RPE cultures.

	Confluent	Confluent (quiescent)	Polarized
Ki-67	89.87 ± 1.72	4.45 ± 0.52	0.09 ± 0.09
p27	0.33 ± 0.19	56.71 ± 6.17	99.68 ± 0.22

### Effect of exogenous BMP-4 treatment on polarized RPE cells

We then evaluated the effect of polarization of the RPE monolayer on PEDF and VEGF secretion after stimulation with an exogenous growth factor. We chose BMP-4 for these studies because BMP-4 plays an important role in RPE development and specification [28,29], is preferentially expressed in RPE in the adult retina [30,31], is over-expressed in RPE in dry AMD [32], and it has been shown to regulated expression of other growth factors including VEGF [27,35].

**Figure 6.**
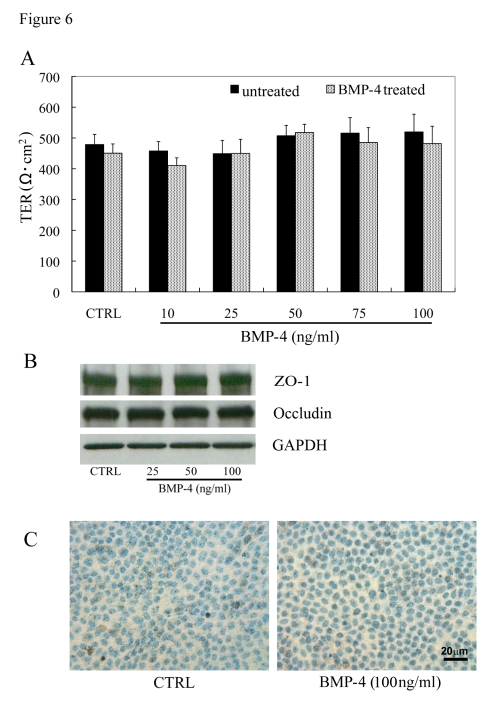
Effect of BMP-4 treatment in highly differenti-ated RPE monolayers. (**A**) Transepithelial resistance (TER) of polarized human RPE monolayers and effect of rhBMP-4 treatment. TER values in human RPE monolayers, maintained for 1 month in 1% FBS-containing medium, averaged 490 ±17 Ω. cm^2^ (mean ± SEM, n=48). The TER measurements in polarized RPE cells exposed to rhBMP-4 treatment for 24 h showed no significant difference (P>0.05) versus untreated controls (n=9/group). (**B**) Expression levels of tight junction proteins, ZO-1 and occludin were not significantly different between the BMP-4 treated and the untreated control groups. (**C**) No significant cell death was observed by TUNEL staining in highly polarized RPE cells of both untreated control and 100ng/ml BMP-4 treatment groups.

To ensure that any changes in growth factor  expression or secretion were not a result of BMP-4 induced cytotoxicity, we evaluated the effect of 24-hr exposure of BMP-4, in a dose-response manner, on TER, expression of tight junction proteins, and induction of apoptosis. Exposure of human polarized RPE (n=4) on Transwell filters to BMP-4 (10-100 ng/ml) did not result in any significant change (ANOVA; p=0.74) in TER versus untreated controls (Figure 6A). Similarly, immunoblot analysis showed no change in expression of ZO-1 or occludin in the BMP-4 treated cells vs untreated controls (Figure 6B). Finally, there was no evidence of apoptosis with the highest BMP-4 dose (100 ng/ml) treated monolayers as determined by TUNEL staining (Figure 6C).

### Effect of BMP-4 treatment on VEGF and PEDF secretion in nonpolarized and polarized RPE

**Figure 7.**
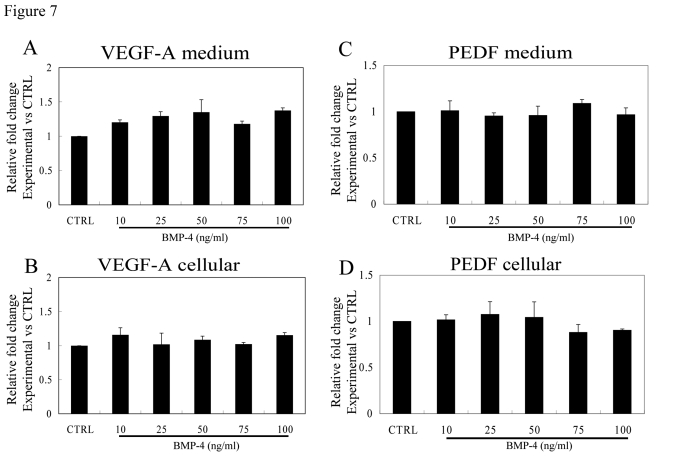
Effect of rhBMP-4 treatment on secretion of VEGF-A and PEDF from nonpolarized RPE cells. Secretion of VEGF-A (**A**) and PEDF (**C**) are presented along with the corresponding cellular VEGF-A (**B**) and cellular PEDF (**D**) from three different donors. Data are presented as fold difference as compared to untreated controls. The cellular concentrations of VEGF-A and PEDF did not differ from untreated controls for the entire BMP-4 concentration range.

The effect of rhBMP-4 (24 hrs; 10-100 ng/ml) on the secretion of VEGF-A and PEDF from non-polarized, confluent human RPE cells was determined in RPE isolated from three individual donors. No significant change in VEGF or PEDF secretion or cellular protein expression was found in non-polarized RPE after treatment with BMP-4 at any of the tested doses (Figure 7).

**Figure 8.**
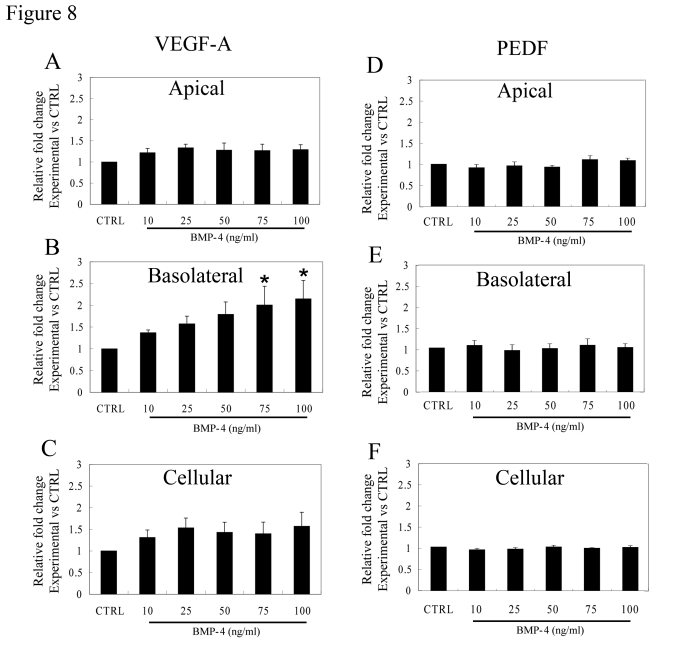
Effect of BMP-4 on VEGF-A and PEDF secretion from polarized RPE. Fold change over control values calculated from ELISA analysis is presented to account for inter donor variations.  (**A**)  The increase in VEGF-A secretion after treatment with BMP-4 from the apical domain was not statistically significant (p>0.05). (**B**)   An increase in VEGF-A secretion from the basolateral domain was found even with the lowest dose used (10ng/ml) which increased further in a dose-dependent fashion. Asterisk indicates that VEGF-A secretion with 75 and 100ng/ml BMP-4 treatment was significantly higher than that of control (p<0.05). (**C**) The cellular levels of VEGF-A were not significantly affected by BMP-4 treatment. (**D, E, F**) No significant change was observed for PEDF secretion either at the apical domain or the basolateral domain and in cellular PEDF levels.  Data are mean±SEM from four different donors.

As was shown earlier in Figure 3, VEGF-A is predominantly secreted from the basolateral domain of polarized RPE monolayers. After 24 hr treatment with BMP-4, secretion of VEGF from the basolateral side of the monolayers remained significantly higher (p<0.01) than that from the apical domain for each BMP-4 concentration ranging from 10 ng/ml to 100 ng/ml (Figure 8). Moreover, there was a dose-dependent increase in basolateral secretion of VEGF that was significant at BMP-4 concentrations of 75 and 100 ng/ml, where it was >2-fold greater than secretion from control polarized monolayers (p<0.05 vs untreated controls, Figure 8B). Importantly, there was no significant increase in apical secretion of VEGF after treatment with BMP-4 (Figure 8A). While cellular VEGF concentrations tended to increase after BMP-4 treatment, these levels did not achieve statistical significance (Figure 8C). In contrast to VEGF, neither cellular PEDF expression, nor secretion from either apical or basolateral domains showed any significant difference after BMP-4 treatment when compared to untreated polarized controls (Figure 8D, E, F).

### BMP-4 effect on VEGF and PEDF gene expression in polarized RPE

**Figure 9.**
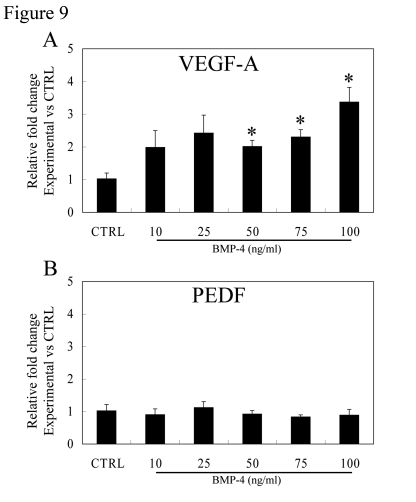
Effect of BMP-4 treatment on gene expression of VEGF-A and PEDF in polarized RPE. Expression of VEGF-A (**A**) and PEDF (**B**) mRNA in polarized fetal RPE cells vs controls was analyzed by real-time PCR. BMP-4 treatment caused an increase in VEGF-A gene expression, especially at 50, 75, and 100ng/ml BMP-4 treatment which was significantly different from controls (p<0.05). PEDF mRNA did not change with BMP-4 dose for the BMP-4 dose range studied.

Figure 9 shows the effect of rhBMP-4 on expression of VEGF-A and PEDF mRNA in polarized RPE monolayers. As compared to untreated controls, VEGF-A mRNA expression showed a significant increase with rhBMP-4 at 50, 75 and 100ng/ml, which was 2.0, 2.3 and 3.4 fold higher (p<0.05 versus untreated controls, respectively) (Figure 9A). Unlike VEGF-A, levels of PEDF mRNA after rhBMP-4 treatments were not significantly different from those of controls (Figure 9B).

## Discussion

We have studied the expression and secretion of the two key growth factors linked to AMD *viz* PEDF and VEGF in confluent human RPE and in highly polarized human RPE monolayers.  Our data show that both PEDF and VEGF are secreted from RPE, with levels of PEDF secretion three orders of magnitude greater than that for VEGF.  Further, in polarized RPE, PEDF was found to be selectively secreted to the apical side while VEGF secretion is basolateral.  Polarization as compared to quiescence was predominantly responsible for regulating growth factor secretion in confluent polarized RPE monolayers. Our studies further showed that BMP-4 induced selective VEGF secretion to the basolateral side of RPE.

Polarization is an essential feature of the differentiated phenotype of the RPE monolayer allowing for attachment to Bruch's membrane, formation of the outer blood-retina-barrier, and specialization of the RPE cells' apical surface for efficient phagocytosis of shed rod outer segments. Furthermore, the RPE cell plays an essential role in the vectorial transport of water, electrolytes and nutrients between the choroid and the neural retina that is also dependent upon the appropriately polarized expression of the relevant integral membrane transporters. Another critical, but less studied function of the RPE layer is the trophic support it provides to the photoreceptors and choroid through the polarized secretion of trophic growth factors such as PEDF and VEGF. In the normal eye, apical secretion of PEDF from the RPE into the interphotoreceptor matrix provides a depot of neurotrophic growth factor support for the photoreceptors, while basal secretion of VEGF from the RPE provides constitutive support for the maintenance of the choriocapillaris [36]. Clearly, trophic growth factors must be secreted within a defined concentration range to be functionally effective.

In disease states such as neovascular AMD and proliferative vitreoretinopathy, there is considerable evidence that dysregulated growth factor expression plays a role in disease pathogenesis. For example, an increase in secretion of VEGF into the pathologic range, with a decrease in secretion of PEDF out of the trophic range, could promote retinal neovascularization while decreasing the support of the photoreceptors [37]. In CNV lesions in AMD, RPE cells become transdifferentiated, lose their polarity and express very high levels of VEGF, thus promoting the development of CNV [38].  Recent reports have confirmed that the primary event in GA is at the level of RPE and that expression and localization of basolateral proteins such as CD63 and MCT3 diminish with the progression of RPE alteration across GA lesions, also suggesting loss of polarity in the late dry form of AMD [24].   Ablonczy et al. [39] suggested that apical PEDF secretion from ARPE-19 cells is important for protection from oxidant induced secretion of VEGF, a mechanism that may be operating in vivo to maintain healthy photoreceptors. In this report we evaluated the hypothesis that polarization of the RPE monolayer is essential for regulating the appropriate level of expression of trophic growth factors such as VEGF and PEDF, without increasing VEGF levels to those needed to induce pathologic angiogenesis.

In this study, the human RPE monolayers in Transwell filters showed well developed epithelial polarity. The monolayer was characterized by the following features: the formation of regular polygonal arrays of cells which increase their pigmentation after cell division, and the presence of tight junction proteins, ZO-1 and occludin. TEM also showed tight-junction complexes, cells with cuboidal to columnar shape and polarized distribution of many organelles. In addition, SEM revealed high  density microvilli akin to resting RPE *in vivo*. The above criteria suggest that our Transwell cell culture model displays classic epithelial polarity. Furthermore, in our study, TER values of polarized RPE cells averaged as high as 500Ω·cm^2^. Other polarized cell culture systems using ARPE-19 cells were found to display morphological features described above, though in most reports TER values less than 100Ω·cm^2^ were found [5,11,40]. Higher TER implied that the cells have well developed tight junctions [41,42], therefore the RPE cells demonstrated prominent polarity. Taken together, our results suggest that the cultured RPE cell preparations behaved similarly to that of differentiated resting RPE *in vivo*.

Several studies on development of well defined polarized culture cell systems exist [4,15,16], but to our knowledge, there is no report on comparison of differences in functional behavior between polarized and nonpolarized human RPE isolated form the same donors. In this study, we at first attempted to evaluate the ability and mode of secretion of PEDF and VEGF-A in both types of RPE cells.  It is noteworthy that among the human RPE cells derived from several donors, those with a higher polarity produced increased amounts of PEDF and VEGF-A than nonpolarized cells as shown by analysis of extracellular medium, cellular protein and cellular mRNA. This indicates that a higher degree of differentiation of RPE cells *in vitro* leads to higher production of PEDF and VEGF. The three orders of magnitude higher levels of expression of PEDF compared to VEGF suggest that PEDF is critical for neurotrophic support of photoreceptors and maintaining an antiangiogenic outer retinal microenvironment, while relatively low levels of basolateral VEGF maintain the choriocapillaris without inducing choroidal neovascularization. The 34 fold increase in PEDF with polarization further supports the importance of RPE polarization in maintenance of this neuroprotective function. It is of interest that neurotrophic PEDF activity was first isolated from conditioned media of polarized RPE [43] and that subsequent secretion of PEDF from RPE also utilized polarized cultures [44].

PEDF expression has also been shown to be regulated during the cell cycle.  Pollina et al. reported that amount of PEDF secretion correlated with cell cycle and secretion was higher in the quiescent stage in fibroblasts [34]. The PEDF promotor activity in fibroblast-like HDF cells was found to be age and cell-cycle dependent [45].  In this study, the difference in the amount of PEDF secretion is also reduced in quiescent vs proliferating RPE, however, the extent of this effect is not significant compared to the 34-fold difference found between polarized vs nonpolarized cells. This finding suggests that the increase in PEDF secretion in the highly differentiated monolayer arose primarily induction of polarity consistent with our hypothesis. The detailed mechanism of higher secretory ability of polarized RPE remains to be elucidated, however, the polarized culture system is a good mimic of the resting RPE and indication of increased secretion may be related to maturation of human RPE cell secretory pathways [15,16].

Our studies confirm and extend the findings from previous studies [15,40], that VEGF-A is preferentially secreted into the basal side of unstimulated RPE. This property of polarized secretion may be necessary so that RPE cells can modulate the homeostasis of the extracellular space around Bruch's membrane and at the same time modulate the density of endothelial cell fenestrations in the choroidal blood supply [46,47]. In contrast to VEGF-A, PEDF in this model is secreted more into the apical side of the RPE and this polarized secretion pattern is consistent with the *in vivo* PEDF expression pattern [36,48]. The amount of PEDF secretion in this study is higher than that of found in monkey eye [36]. It is of interest that in the monkey model, the authors suggested that polarization of RPE may be an important mechanism that regulates PEDF secretion [36,49]. Increased PEDF secretion from RPE may be necessary for retinal neuroprotection. Indeed Mukherjee et al. [50] showed recently that PEDF produced in the apical media of ARPE-19 cells augmented NPD1-mediated protection. Another interesting feature of our studies was that, although interdonor variations exist with respect to the amount of PEDF and VEGF secretion, the relative  apical/basolateral ratio for both PEDF and VEGF among donors remained remarkably similar.

In further studies to evaluate the functional ability of this polarized RPE culture system to mimic human disease, we evaluated the effect of BMP-4 on growth factor secretion since BMP-4 expression is upregulated  in dry AMD [32]. Our experiments showed that exogenous rhBMP-4 significantly increased basolateral VEGF-A secretion in a dose-dependent manner. We believe that this is the first demonstration of polarized VEGF-A secretion by human RPE upon stimulation with BMP-4. Our results are in agreement with a recent report [27] in which an entirely different protocol for BMP-4 administration to ARPE-19 cells was employed. However, our findings differ from another study [51] in which BMP-4 did not affect VEGF secretion. Clearly, the state of differentiation and polarization of RPE cells influences the effect of exogenous growth factors (such as BMP-4) on RPE secretion of VEGF-A.  In this context, it can be said that to evaluate RPE function with various treatments *in vitro*, polarized RPE might represent the resting RPE more accurately. Since secretion of VEGF-A was found to be upregulated by BMP-4, we evaluated the effect of the treatment of human RPE with noggin, a BMP-4 antagonist [52]. Noggin significantly inhibited VEGF-A secretion by about 40% under our experimental conditions thereby confirming a role for BMP-4 in stimulating VEGF-A secretion (p<0.05; data not shown). Recently, it was shown that in AMD patients with CNV, the RPE in CNV lesions showed essentially absent immunohistochemical levels of expression of BMP-4 suggesting that lack of BMP-4 may be permissive for pathologic angiogenesis [53]. It is likely that other factors, such as inflammation regulation RPE expression levels of BMP-4, and that the very high, pathologic levels of VEGF found in nonpolarized, transdifferentiated RPE found in CNV lesions are regulated by factors other than BMP-4 [53].

In conclusion, our data show that polarity is an important determinant of the level of PEDF and VEGF secretion in RPE and support the contention that loss of polarity of RPE in AMD results in marked loss of neurotrophic and vascular support for the retina potentially leading to photoreceptor loss and blindness.

## Materials and methods

RPE cell culture . All experiments and procedures were conducted in compliance with the Declaration of Helsinki.  RPE cells were isolated from human fetal eyes were cultured as previously described [3,54]. Confluent cell cultures from passages 2 to 4 were used. RPE were cultured under 3 conditions for comparison: [1]. confluent (1day culture in 10% fetal bovine serum (FBS) in Dulbecco's minimum Eagle's medium (DMEM) followed by 1% FBS for three days in 6 well plate); [2] confluent-quiescent (cultured for additional 7 days in 1% FBS in 6-well plates); and [3] highly differentiated polarized RPE (grown on Transwell filters for a period of more than 1 month in 1% FBS).

Human RPE monolayer cultures on Transwell filters. Highly differentiated fetal human RPE cells were grown utilizing the protocol of Maminishkis et al.[15]with some modifications [3]. Briefly, primary cultures of human fetal RPE cells from multiple donors were trypsinized and resuspended in media supplemented with 10% FBS. Approximately 1.0×10^5^ human RPE cells/cm^2^ were seeded on fibronectin-coated Transwell filters (12 mm internal diameter; 0.4 μm pore size;Corning Costar). RPE cells were cultured on the filtersin 10% FBS containing medium for 1 day and in 1% FBS thereafter for one month. This resulted in the formation of differentiated polarized monolayers, with the apical domain corresponding to the retinal facing side of the RPE monolayer and basolateral domain corresponding to the choroidal facing side of the RPE monolayer. One milliliter of serum free culture medium was introduced to both apical and basolateral chambers in experiments to determine secretion.

Measurement of Transepithelial resistance (TER). TER of RPE monolayers grown on Transwells was measured with an EVOM epithelial tissue voltohmmeter (World Precision Instruments) as described [40]. All TER measurements were made in a cell culture hood within 3 min of removal of Transwells from the incubator, and the average temperature at the time of measurement was 32.2 ±1.85ºC. Net TERs were calculated by subtracting the value of a blank, fibronectin-coated Transwell filter without cells from the experimental value. Final resistance-area products (Ω·cm^2^) were obtained by multiplication with the effective growth area [40].

Confocal immunofluorescence. The morphologic features of polarization were visualized by immunolocalization of ZO-1 and occludin to the junctional complex, and apical localization of Na/K- ATPase [3,40]. Cultures were also evaluated for cell cycle status by assessing expression of Ki-67 and p27. RPE monolayers were fixed in 2% paraformaldehyde followed by blocking with in 5% BSA  before incubating with ZO-1 rabbit polyclonal antibody (1:100 dilution, Zymed), rabbit polyclonal anti-occludin (1:100, Zymed), monoclonal antibody labeling Na/K- ATPase (1 μg/ml, Upstate), mouse monoclonal antibody against Ki-67 (1:100, Millipore) and mouse monoclonal antibody against p27 (1:40, Novocastra Laboratories) at 4°C overnight. The cells were washed and incubated with FITC conjugated anti-rabbit or anti-mouse secondary antibody (Jackson Labs) for 30 min. After the immunostaining procedure, membranes were removed from the inserts with a fine, sharp, sterile razor blade and mounted on a glass slide with fluorescent mounting medium containing 4',6-diamidino-2-phenylindole (DAPI; Vector Laboratories) and viewed on an LSM 510 laser-scanning microscope (Carl Zeiss).

Confluence, polarity and cell proliferation status. To differentiate between effects of cell proliferation and polarity on extent of growth factor secretion, cell cycle status was evaluated in RPE cultured in three different ways. These consisted of [1] confluent (1day culture in 10% FBS in DMEM followed by 1% FBS for three days on glass chamber slide), [2] confluent-quiescent (cultured for additional 7 days on glass chamber slide), and [3] highly differentiated polarized RPE (grown on Transwell filters for a > 1 month in 1% FBS). Staining for p27 (highly expressed in quiescent cells) and Ki-67 (highly expressed in dividing cells) was performed and relative proportions of p27 and Ki-67 positive cells were counted from confocal images. In addition, media from the above three culture conditions was analyzed for VEGF and PEDF secretion.

Scanning Electron Microscopy. The monolayer of RPE was fixed in half strength Karnovsky's fixative and then postfixed in 1% osmium tetroxide.  After a cacodylate buffer rinse, the monolayers were dehydrated through an alcohol series then transferred from 100% ethanol to 100% hexamethyldisilasane (HMDS). After two changes in HMDS, the monolayers were allowed to air dry for 24 h. The membranes with attached monolayers were next mounted on to stubs and coated with gold and palladium on a sputter-coater.  The cells were imaged with a JEOL JSM 6390 LV Scanning Electron Microscope (filament voltage at 15 KV).

Transmission electron microscopy. RPE monolayers were fixed in half strength Karnovsky's fixative for 24 h at 4^0^C. The cell monolayers were then postfixed in 1% osmium tetroxide for 2h on ice. The samples were dehydrated in ethyl alcohol and then infiltrated in Eponate prior to embedding. Ultrathin sections were cut at a thickness of 70nm and stained with uranyl acetate and lead citrate. Sections were examined on a JEOL JEM 2100 electron microscope.

BMP-4 treatments. In both non-polarized and polarized cells,  the RPE culturemedium was switched to 0% FBS overnight and then replaced with fresh 0% FBS  culture medium for 24 h. Recombinant human BMP-4 (0, 10, 25, 50, 75, 100ng/ml, R&D Systems) was introduced to the medium in the non-polarized cells, and in the medium on both sides (apical and basolateral) of the membrane for 24 h in the polarized cells. After the incubation period, the extracellular mediumwas collected for protein secretion analysis, and the cellswere used for mRNA and protein quantification studies. In our studies, BMP-4 was introduced to RPE Transwell filters from both the apical and basolateral compartments each maintained in a volume of 1ml of the incubation medium. To exclude the possibility of this modification influencing the secretion properties as compared to the previously used 0.5ml apical, 1.5ml basolateral medium protocols [15], TER and PEDF secretion were measured in separate experiments of RPE Transwells maintained in apical/basolateral volume combinations of 0.5ml/0.5ml, 0.5ml/1.0ml, 0.5ml/1.5ml, 1.0ml/1.0ml incubation media. No significant change in TER or PEDF secretion among groups could be detected under these experimental conditions (data not shown) and subsequent experiments were all performed using a 1.0ml/1.0ml incubation medium.

Enzyme-linked immunosorbent assay (ELISA). In non-growth factor treated cells, and at the end of experiments in which cells were treated with BMP-4, the extracellular medium fromcontrol and treated non-polarized RPE groups and the medium fromthe apical and basal compartments of the highly polarized RPEgroups were collected and stored at -80°C until furtheranalysis. Levels of VEGF-A (Quantikine; R&D Systems) and PEDF (BioProducts) in the medium was measured accordingto the manufacturers' protocols. In separate experiments,cellular levels of VEGF-A and PEDF were measured asdescribed previously [40]. Data derived from standard curves wereexpressed as picograms per milliliter for the two growth factors secretedinto medium, and as relative difference (*x*-fold) in growth factorprotein relative to the untreated control in cellular lysates.

Western blot analysis for ZO-1 and occludin. After treatment with BMP-4, the cell lysates weresubjected to Western blot analysis as previously described [54].  Primary antibodies used were ZO-1 rabbit polyclonal antibody (1:1000 dilution; Zymed) and anti-occludin rabbit polyclonal antibody (1:500 dilution; Zymed).After incubation with horseradish peroxidase-conjugatedanti-rabbit secondary antibody (Vector Laboratories),protein bands were detected by chemiluminescence (Pierce). To verify equal loading, membranes were reprobed with GAPDH.

Real-time RT-PCR. Total RNA was isolated (TRIzol extraction protocol; Invitrogen), and treated with DNase (Ambion)to remove contaminating genomic DNA. Reverse transcriptionwas performed with 1 μg total RNA, oligo(dT)_15_ primer,and AMV reverse transcriptase according to the manufacturer'sprotocol (Promega). The PCR experiments were performed on a thermocycler (model LC 480 light cycler; RocheDiagnostics), with SYBR Green (Roche Diagnostics)as the interaction agent. Each 20 μL PCR mix contained 5 μLcDNA template, 10 μL SYBR Green PCR master mix, and 0.5μM of each gene-specific primer. Quantification of mRNA was normalized with *GAPDH* as thehousekeeping gene. The specificity of the PCR amplificationproducts was checked by performing dissociation melting curveanalysis and by 1% agarose gel electrophoresis. Reaction conditionswere as follows: 5 min at 95°C followed by 45 cyclesof 10 sec at 95°C, 20 sec at 55°C, and 20 secat 72°C. The sequences of primers used for human VEGF-Awere forward: 5'-TCT TCA AGC CAT CCT CTG TG-3', reverse: 5'-ATC CGC ATA ATC TGC ATG GT-3'; PEDF forward: 5'-ACG CTA TGG CTT GGA TTC AG-3', reverse: 5'-GGT CAA ATT CTG GGT CAC TTT C-3'. Relativemultiples of changes in mRNA expression were determined by calculationof -2^∆∆*C*T^. Results are reported as the mean difference inrelative multiples of change in mRNA expression ± SEM.

TUNEL Staining. Apoptosis was detected by the terminal deoxynucleotidyltransferase (TdT)-mediated dUTP-biotin nick end-labeling (TUNEL)method according to the manufacturer's protocol (ApopTagperoxidase in situ apoptosis detection kit; Chemicon). Briefly, cells were fixed in 3% paraformaldehyde solutionand rinsed with PBS. After treatment with 3% H_2_O_2_ at room temperaturefor 5 min, the cells were incubated with TdT enzyme for1 h at 37°C in a humidified chamber. The digoxigenin (DIG) labelednucleotides incorporated into DNA breaks were detected by applyinganti-digoxigenin conjugate and peroxidase substrate.

Statistical analysis. All values were expressed as mean± S.E.M. Differences between two groups were analyzed by paired t-test, and those among multiple groups were analyzed by analysis of variance (ANOVA) followed by Sheffe's test. Differences with a P value of less than 0.05 were considered to be significant.

## References

[1] (1972). Hogan MJ. Role of the retinal pigment epithelium in macular disease. Trans Am Acad Ophthalmol Otolaryngol.

[2] Sheedlo HJ, Li L, Turner JE (1992). Effects of RPE-cell factors secreted from permselective fibers on retinal cells in vitro. Brain Res.

[3] Sonoda S, Spee C, Barron E, Ryan SJ, Kannan R, Hinton DR (2009). A protocol for the culture and differentiation of highly polarized human retinal pigment epithelial cells. Nature Protocols.

[4] Hu J, Bok D (2001). A cell culture medium that supports the differentiation of human retinal pigment epithelium into functionally polarized monolayers. Mol Vis.

[5] Holtkamp GM, Van Rossem M, de Vos AF, Willekens B, Peek R, Kijlstra A (1998). Polarized secretion of IL-6 and IL-8 by human retinal pigment epithelial cells. Clin Exp Immunol.

[6] Blaauwgeers HG, Holtkamp GM, Rutten H (1999). Polarized vascular endothelial growth factor secretion by human retinal pigment epithelium and localization of vascular endothelial growth factor receptors on the inner choriocapillaris. Evidence for a trophic paracrine relation. Am J Pathol.

[7] Ban Y, Rizzolo LJ (1997). A culture model of development reveals multiple properties of RPE tight junctions. Mol Vis.

[8] Dunn KC, Aotaki-Keen AE, Putkey FR, Hjelmeland LM (1996). ARPE-19, a human retinal pigment epithelial cell line with differentiated properties. Exp Eye Res.

[9] Marin-Castano ME, Csaky KG, Cousins SW (2005). Nonlethal oxidant injury to human retinal pigment epithelium cells causes cell membrane blebbing but decreased MMP-2 activity. Invest Ophthalmol Vis Sci.

[10] Handa JT, Reiser KM, Matsunaga H, Hjelmeland LM (1998). The advanced glycation endproduct pentosidine induces the expression of PDGF-B in human retinal pigment epithelial cells. Exp Eye Res.

[11] Luo Y, Zhuo Y, Fukuhara M, Rizzolo LJ (2006). Effects of culture conditions on heterogeneity and the apical junctional complex of the ARPE-19 cell line. Invest Ophthalmol Vis Sci.

[12] Rahner C, Fukuhara M, Peng S, Kojima S, Rizzolo LJ (2004). The apical and basal environments of the retinal pigment epithelium regulate the maturation of tight junctions during development. J Cell Sci.

[13] Peng S, Rahner C, Rizzolo LJ (2003). Apical and basal regulation of the permeability of the retinal pigment epithelium. Invest Ophthalmol Vis Sci.

[14] Geisen P, McColm JR, King BM, Hartnett ME (2006). Characterization of barrier properties and inducible VEGF expression of several types of retinal pigment epithelium in medium-term culture. Curr Eye Res.

[15] Maminishkis A, Chen S, Jalickee S (2006). Confluent monolayers of cultured human fetal retinal pigment epithelium exhibit morphology and physiology of native tissue. Invest Ophthalmol Vis Sci.

[16] Shi G, Maminishkis A, Banzon T (2008). Control of Chemokine Gradients by the Retinal Pigment Epithelium. Invest Ophthalmol Vis Sci.

[17] Ohno-Matsui K, Morita I, Tombran-Tink J (2001). Novel mechanism for age-related macular degeneration: an equilibrium shift between the angiogenesis factors VEGF and PEDF. J Cell Physiol.

[18] Sakamoto T, Sakamoto H, Hinton DR, Spee C, Ishibashi T, Ryan SJ (1995). In vitro studies of human choroidal endothelial cells. Curr Eye Res.

[19] Miller H, Miller B, Ryan SJ (1986). The role of retinal pigment epithelium in the involution of subretinal neovascularization. Invest Ophthalmol Vis Sci.

[20] Shweiki D, Itin A, Soffer D, Keshet E (1992). Vascular endothelial growth factor induced by hypoxia may mediate hypoxia-initiated angiogenesis. Nature.

[21] Adamis AP, Miller JW, Bernal MT (1994). Increased vascular endothelial growth factor levels in the vitreous of eyes with proliferative diabetic retinopathy. Am J Ophthalmol.

[22] Dawson DW, Volpert OV, Gillis P (1999). Pigment epithelium-derived factor: a potent inhibitor of angiogenesis. Science.

[23] McLeod DS, Grebe R, Bhutto I, Merges C, Baba T, Lutty GA (2009). Relationship between RPE and choriocapillaris in age-related macular degeneration. Invest Ophthalmol Vis Sci.

[24] Vogt SD, Curcio CA, Wang L, Li CM, McGwin G, Medeiros NE Jr, Philp NJ, Kimble JA, Read RW (2009). Altered retinal pigment epithelium morphology is associated with decreased expression of complement regulatory protein CD46 and ion transporter MCT3 in geographic atrophy of age-related maculopathy. Invest Ophthalmol Vis Sci.

[25] Bian ZM, Elner SG, Elner VM (2007). Regulation of VEGF mRNA expression and protein secretion by TGF-beta2 in human retinal pigment epithelial cells. Exp Eye Res.

[26] Zamiri P, Masli S, Streilein JW, Taylor AW (2006). Pigment epithelial growth factor suppresses inflammation by modulating macrophage activation. Invest Ophthalmol Vis Sci.

[27] Vogt RR, Unda R, Yeh LC, Vidro EK, Lee JC, Tsin AT (2006). Bone morphogenetic protein-4 enhances vascular endothelial growth factor secretion by human retinal pigment epithelial cells. J Cell Biochem.

[28] Muller F, Rohrer H, Vogel-Hopker A (2007). Bone morphogenetic proteins specify the retinal pigment epithelium in the chick embryo. Development.

[29] Furuta Y, Piston DW, Hogan BL (1997). Bone morphogenetic proteins (BMPs) as regulators of dorsal forebrain development. Development.

[30] Mathura JR Jr, Jafari N, Chang JT (2000). Bone morphogenetic proteins-2 and -4: negative growth regulators in adult retinal pigmented epithelium. Invest Ophthalmol Vis Sci.

[31] Wordinger RJ, Clark AF (2007). Bone morphogenetic proteins and their receptors in the eye. Exp Biol Med (Maywood).

[32] Zhu D, Wu J, Spee C, Ryan SJ, Hinton DR (2009). BMP4 mediates oxidative stress-induced retinal pigment epithelial cell senescence and is over expressed in age-related macular degeneration. J Biol Chem.

[33] Pignolo RJ, Francis MK, Rotenberg MO, Cristofalo VJ (2003). Putative role for EPC-1/PEDF in the G0 growth arrest of human diploid fibroblasts. J Cell Physiol.

[34] Pollina EA, Legesse-Miller A, Haley EM, Goodpaster T, Randolph-Habecker J, Coller HA (2008). Regulating the angiogenic balance in tissues. Cell Cycle.

[35] Rothhammer T, Bataille F, Spruss T, Eissner G, Bosserhoff AK (2007). Functional implication of BMP4 expression on angiogenesis in malignant melanoma. Oncogene.

[36] Becerra SP, Fariss RN, Wu YQ, Montuenga LM, Wong P, Pfeffer BA (2004). Pigment epithelium-derived factor in the monkey retinal pigment epithelium and interphotoreceptor matrix: apical secretion and distribution. Exp Eye Res.

[37] (2001). Gao G, Li Y, Zhang D, Gee S, Crosson C, Ma J. Unbalanced expression of VEGF and PEDF in ischemia-induced retinal neovascularization. FEBS Lett.

[38] (1996). Lopez PF, Sippy BD, Lambert HM, Thach AB, Hinton DR. Transdifferentiated retinal pigment epithelial cells are immunoreactive for vascular endothelial growth factor in surgically excised age-related macular degeneration-related choroidal neovascular membranes. Invest Ophthalmol Vis Sci.

[39] Ablonczy Z, Prakasam A, Fant J, Fauq A, Crosson C, Sambamurti K (2009). Pigment epithelium-derived factor maintains retinal pigment epithelium function by inhibiting vascular endothelial growth factor-R2 signaling through gamma-secretase. J Biol Chem.

[40] Kannan R, Zhang N, Sreekumar PG (2006). Stimulation of apical and basolateral VEGF-A and VEGF-C secretion by oxidative stress in polarized retinal pigment epithelial cells. Mol Vis.

[41] Quinn RH, Miller SS (1992). Ion transport mechanisms in native human retinal pigment epithelium. Invest Ophthalmol Vis Sci.

[42] Quinn RH, Quong JN, Miller SS (2001). Adrenergic receptor activated ion transport in human fetal retinal pigment epithelium. Invest Ophthalmol Vis Sci.

[43] Tombran-Tink J, Johnson LV (1989). Neuronal differentiation of retinoblastoma cells induced by medium conditioned by human RPE cells. Invest Ophthalmol Vis Sci.

[44] Tombran-Tink J, Shivaram SM, Chader GJ, Johnson LV, Bok D (1995). Expression, secretion, and age-related downregulation of pigment epithelium-derived factor, a serpin with neurotrophic activity. J Neurosci.

[45] Kojima T, Nakahama K, Yamamoto K, Uematsu H, Morita I (2006). Age- and cell cycle-dependent changes in EPC-1/PEDF promoter activity in human diploid fibroblast-like (HDF) cells. Mol Cell Biochem.

[46] Roberts WG, Palade GE (1995). Increased microvascular permeability and endothelial fenestration induced by vascular endothelial growth factor. J Cell Sci.

[47] Yokomori H, Oda M, Yoshimura K (2003). Vascular endothelial growth factor increases fenestral permeability in hepatic sinusoidal endothelial cells. Liver Int.

[48] Karakousis PC, John SK, Behling KC (2001). Localization of pigment epithelium derived factor (PEDF) in developing and adult human ocular tissues. Mol Vis.

[49] Pfeffer BA, Becerra SP, Borst DE, Wong P (2004). Expression of transthyretin and retinol binding protein mRNAs and secretion of transthyretin by cultured monkey retinal pigment epithelium. Mol Vis.

[50] Mukherjee PK, Marcheselli VL, Barreiro S, Hu J, Bok D, Bazan NG (2007). Neurotrophins enhance retinal pigment epithelial cell survival through neuroprotectin D1 signaling. Proc Natl Acad Sci U S A.

[51] Nagineni CN, Samuel W, Nagineni S (2003). Transforming growth factor-beta induces expression of vascular endothelial growth factor in human retinal pigment epithelial cells: involvement of mitogen-activated protein kinases. J Cell Physiol.

[52] Balemans W, Van Hul W (2002). Extracellular regulation of BMP signaling in vertebrates: a cocktail of modulators. Dev Biol.

[53] Zhu DH, Deng X, Xu J, Hinton DR (2009). What determines the switch between atrophic and neovascular forms of age related macular degeneration? - the role of BMP4 induced senescence. Aging.

[54] Sreekumar PG, Kannan R, Yaung J, Spee CK, Ryan SJ, Hinton DR (2005). Protection from oxidative stress by methionine sulfoxide reductases in RPE cells. Biochem Biophys Res Commun.

